# Unlocking the access to oxidized coenzyme A via a single-step green membrane-based purification

**DOI:** 10.1038/s41598-022-17250-8

**Published:** 2022-07-29

**Authors:** Louis M. M. Mouterde, Gaëlle Willig, Maxime M. J. Langlait, Fanny Brunois, Morad Chadni, Florent Allais

**Affiliations:** URD Agro-Biotechnologies Industrielles (ABI), CEBB, AgroParisTech, 51110 Pomacle, France

**Keywords:** Biocatalysis, Biosynthesis

## Abstract

A new membrane-based strategy to purify oxidized coenzyme A ((CoAS)_2_) from adenosine triphosphate (ATP), adenosine diphosphate (ADP) and adenosine monophosphate (AMP) has been developed. Commercially available membranes were screened and studied (permeate flux and overall compounds retention) which allowed the identification of one efficient membrane (GK from Suez Water Technologies & Solutions). Different total compounds concentrations solutions were used in the system in order to find the following working conditions: 4 bars with a total compounds solution of 5.19 g L^−1^. Applying these conditions to a dia-filtration set-up allowed us to reach 68% pure (CoAS)_2_ in 4.8 diafiltration volumes (DV) and a 95% (CoAS)_2_ purity can be predicted in 8.5 DV. A comparative study of green metrics—i.e. process mass index (PMI)—of the classic chromatography vs the membrane-based one demonstrated the great advantages of the latter in terms of sustainability. This strategy unlocks the access to the essential and central cofactor that is coenzyme A.

In the last decades, industrial biotechnologies have experienced a strong and ever-expanding development to offer sustainable alternatives to chemical processes that are not always respectful of the environment and most often dependent on fossil resources. Fermentation has been shown to be effective for the production of valuable products such as biofuels, therapeutic molecules or food additives^1–10^. However, it can suffer from some limitations such as substrate/product working concentrations (e.g., toxicity towards the microorganism, transfer of the substrate/product through the microorganism membrane), fermentation conditions or downstream processing (e.g., presence of biomass, primary and secondary metabolites). In vitro biocatalysis is a good strategy to implement when these limitations become insuperable.

The great advances achieved in protein engineering and enzyme immobilization allowed the development of robust systems in pharmaceutical, food processing or detergent industries^11–23^. However, enzymes that lead to such systems are very rarely coenzyme-dependent, mainly due to the high commercial cost of these molecules. This is the case for coenzyme A (CoA*, ca.* > $2000/gram) which is involved in many metabolic pathways such as fatty acid synthesis and degradation, amino-acid degradation or oxidation of pyruvate in the Krebs cycle. All genome sequenced to date encode enzymes that necessarily require CoA which represents 4% of known enzymes^24^. These are all potential enzymatic tools that cannot be exploited in vitro systems. It is especially important as alternative biocatalytic C–C bonds forming methods (identified in many metabolic pathways involving CoA) are needed to substitute classical chemical methods, such as aldol reactions or Claisen condensations, that involves toxic and non-sustainable chemicals/solvents. Although gram-scale in vivo and in vitro production methods have been developed to increase the availability of CoA^25–32^, they did not sufficiently lower the process cost mainly due to the expensive purification methods required^33^. Indeed, the latter consist in using different ion exchange chromatography steps and charcoal adsorption/desorption in order to isolate CoA from cellular materials and/or metabolites/coproducts such as adenosine triphosphate (ATP), adenosine diphosphate (ADP) and adenosine monophosphate (AMP)^26,32,34–36^ (Fig. 1). The ion exchange chromatography used to separate these adenosine-based compounds is particularly challenging due to the very similar ionic profile of the molecules. Such method has also been used to purify oxidized CoA ((CoAS)_2_) which present the great advantage of being highly stable compared to CoA and that can give access to the latter through a trivial disulfide bridge reduction^32^.

**Figure 1:**
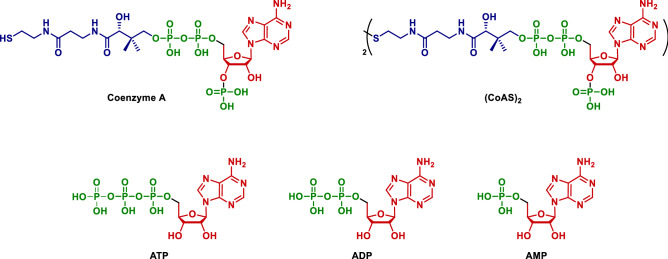
Structures of the coenzyme A, (CoAS)_2_, ATP, ADP and AMP.

In this work, an alternative separation method that do not require additional salts other than the ones needed for the biocatalytic production of (CoAS)_2_, solvent nor chemical compounds, and that takes advantage of both the differences in molecular weights between the reaction mixture products and the stability of (CoAS)_2_, has been investigated^32,36^. According to the aforementioned considerations, a cross-flow dia-ultrafiltration was implemented to efficiently and readily separate (CoAS)_2_ from ATP, ADP and AMP. It must be noted that this strategy would allow an affordable access to (CoAS)_2_ which can only be found nowadays at a very high-cost (*ca.* > $30,000/gram).

## Results

### Membrane selection

**Figure 2:**
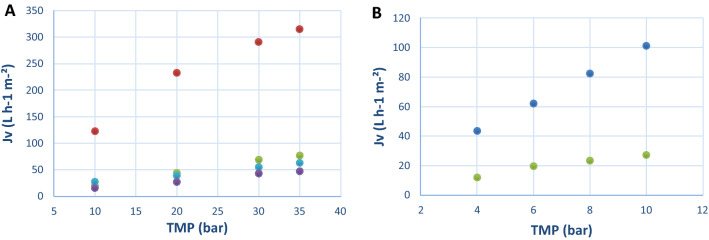
Effect of pressure (TMP) on permeate flux (J_v_) for membranes screened with model solution. (**A**) () NP010, () GE, () 7450, () 7470 PHT. (**B**) () GK, () GR95PP.

**Figure 3:**
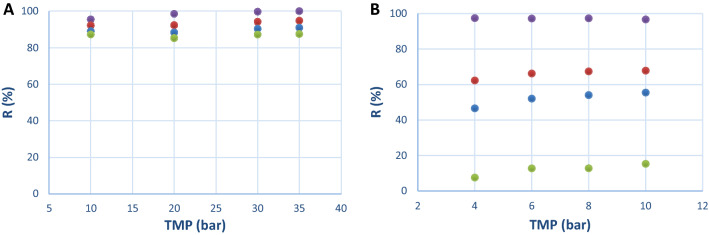
Effect of pressure (TMP) on the compounds’ retention for (**A**) NP010 and (**B**) GK membranes with model solution. () (CoAS)_2_, () ATP, () ADP, () AMP.

The first step of the study consisted in a screening of different membranes using a model solution composed of (CoAS)_2_, ATP, ADP and AMP to evaluate their ability to retain (CoAS)_2_ and to discard ATP, ADP and AMP in a cross-flow diafiltration setup, the molecular weight of the target molecule being 1533 g mol^−1^ and the one of ATP, ADP and AMP being 507, 427 and 347 g mol^−1^, respectively. A molecular weight cut-off (MWCO) from 720 to 2000 Da was selected which allowed us to assess 7 different membranes for the study: NP010, GR95PP, 7450, 7470 PHT, GE and GK. It has to be noted that, according to the data in Table 4, no correlation was observed between water permeabilities (measured at a pressure of 4 bars), MWCO and materials of the different membranes. The effect of the pressure on permeate flux for the different membranes was evaluated using the model solution (AMP (0.04 g L^−1^), ADP (0.52 g L^−1^), ATP (0.15 g L^−1^) and (CoAS)_2_ (0.29 g L^−1^) with a total concentration of 1.00 g L^−1^. It was observed for all membranes that the higher the pressure, the faster the flow is in the tested pressure range. As for the permeate flux, it appeared that NP010 and GK were the most efficient membranes in the tested pressure range (Fig. 2). It has to be noted that due to the design of our screening system, the maximum pressure applied to the membrane was not the highest preconized by the manufacturer. However, it did not affect our study as the best results regarding retention of (CoAS)_2_ and rejection of ATP, ADP and AMP were observed at the lowest pressures^37^. Indeed, the experimental data show that the retention of all the compounds is higher as the pressure rise which can be due to the evolution of polarization concentration or the pore sizes. NP010 showed a high retention profile for all compounds which was not suitable for the purification of (CoAS)_2_, whereas GK membrane presented high retention capacity for (CoAS)_2_, acceptable for ATP and ADP and low for AMP (Fig. 3). These results can be explained by the difference in pore sizes (MWCO of 1000 and 3500 Da for NP010 and GK respectively) and membrane charges—even though no detail is given for the material of GK (modified polyamide), a difference in the isoelectric points (2.5 and 3.5 for NP010 and GK respectively) show that the two membrane have different charges. Overall, the best compromise between permeate flux and retention profile was obtained for GK membrane at 4 bars, with a retention of 97% for (CoAS)_2_, 62% for ATP, 47% for ADP and 8% for AMP and a flux of 43.62 L.h^-1^.m^-2^. The latter was therefore chosen for the rest of the study.

### Influence of the solution concentration on performance parameters

**Figure 4:**
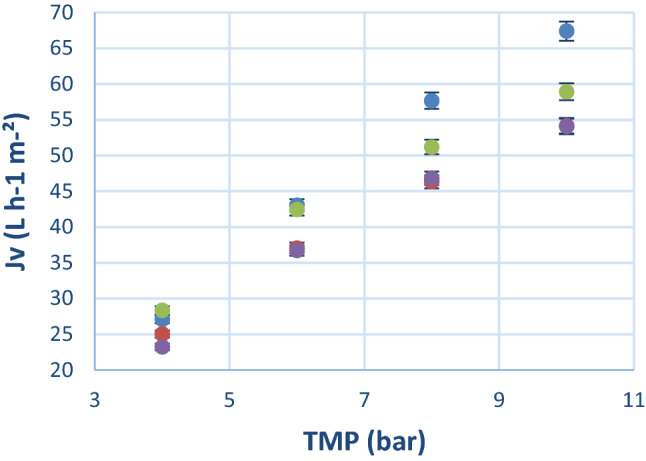
Effects of concentrations on permeate flux (Jv) as a function of transmembrane pressure (TMP). () 1.51 g L^−1^, () 2.43 g L^−1^, () 5.19 g L^−1^, () 10.71 g L^−1^.

The influence of the total compounds solution concentration on the permeate flux and retention profile of the GK membrane was then evaluated in order to intensify the process and reach high productivity rate while decreasing water consumption. A stock solution of (CoAS)_2_, ATP, ADP and AMP, obtained by enzymatic synthesis, was therefore used to give access to solutions at 1.51, 2.43, 5.19 and 10.71 g L^−1^ of total compounds^32^. These concentrations were chosen to increase dry matter in solution without applying a too large concentration polarization to the system. As expected, a decrease in the permeate flux was observed as the concentration increases. It has to be noted that the 10.71 g/L concentration does not follow this tendency since the experiment was carried out with a new flat sheet. Indeed, it is well known that significant variability in term of permeability is observed between different flat sheets and good repeatability can only be observed when using spiral membrane. However, although this diminution is relatively significant at high pressure (10 bars), it is negligible at our optimum pressure of 4 bars (Fig. 4). Moreover, (CoAS)_2_ discrimination vs. ATP and ADP increase as the solution concentration increase. However, it was observed that (CoAS)_2_ retention decreased as the solution concentration increased with an important gap between 5.19 and 10.71 g L^−1^ (Table 1).

**Table 1:** Discrimination profile and (CoAS)_2_ losses at 4 bars.

Concentration (g L^−1^)	(CoAS)_2_ vs. ATP (%)	(CoAS)_2_ vs. ADP (%)	(CoAS)_2_ vs. AMP (%)	(CoAS)_2_ retention (%)
1.51	21.12 ± 0.09	46.18 ± 0.31	84.67 ± 1.16	97.87 ± 0.15
2.43	22.92 ± 0.22	43.89 ± 0.89	80.33 ± 0.50	98.02 ± 0.20
5.19	29.13 ± 0.08	49.73 ± 0.21	77.73 ± 0.54	97.23 ± 0.09
10.71	33.07 ± 0.41	54.53 ± 0.03	70.29 ± 0.64	92.00 ± 0.43

These data allowed us to predict (CoAS)_2_ purity and losses during a diafiltration using the general formula (Eq. 9) and therefore identify the best compromised concentration. A diafiltration volume of 3 was chosen to compare the different conditions. The predicted losses remained below 10% for the 1.51, 2.43 and 5.19 g L^−1^ concentrations, and significantly dropped for the 10.71 g L^−1^ concentration (21%) with no important gain in term of purity (Table 2). By correlating permeate flux (Jv) (Fig. 4) and predicted (CoAS)_2_ losses/purity, the best compromised concentration 5.19 g L^−1^ was chosen to perform the diafiltration.

**Table 2:** Predicted (CoAS)_2_ losses at 4 bars and diafiltration volume 3.

Total compound solution concentration (g L^−1^)	Initial (CoAS)_2_ concentration (g L^−1^)	Final (CoAS)_2_ purity (%)	Total (CoAS)_2_ losses (%)	Final (CoAS)_2_ concentration (g L^−1^)
1.51	0.58	68	6	0.55
2.43	0.94	67	6	0.88
5.19	2.02	71	8	1.86
10.71	4.17	74	21	3.29

### Purification of oxidized coenzyme A via diafiltration process

**Figure 5:**
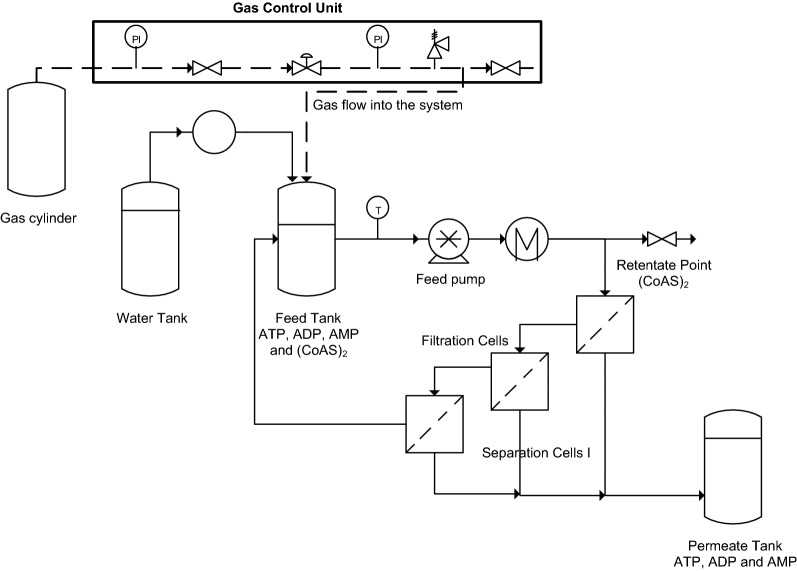
Experimental filtration set-up used for diafiltration experiments.

**Figure 6:**
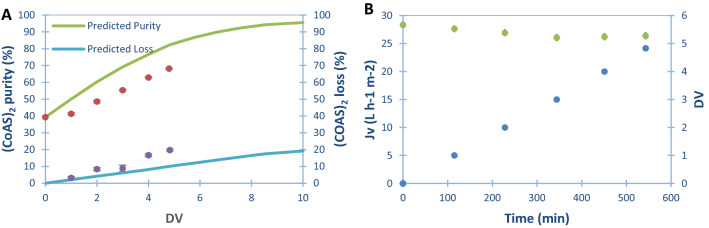
(**A**) Evolution of (CoAS)_2_ purity () and loss () as a function of diafiltration volume (DV) during dia-ultrafiltration process with 5.19 g L^−1^ solution. (**B**) Evolution of permeate flux (Jv) () and diafiltration volume (DV) () as a function of time, with 5.19 g L^−1^ solution.

Total compounds solution concentration of 5.19 g L^−1^ was therefore used in a diafiltration set-up in order to purify (CoAS)_2_ (Fig. 5). A diafiltration volume of 5 was determined to be necessary to reach a purity ≥ 85%. However, the technical constraint of the device forced us to apply a DV of 4.8. The evolution of (CoAS)_2_ purity and loss as a function of diafiltration volume (DV), as well as the evolution of permeate flux (Jv) and diafiltration volume (DV) as a function of time, were assessed (Fig. 6). A poor loss of flux (< 7%) and no significant fouling were observed during the dia-ultrafiltration, by comparing water permeabilities before and after diafiltration, which suggests that not only this technology is very well adapted to this type of solutions, but also that the membrane can be recycled and used for several runs. A lower than expected (CoAS)_2_ purity of 68% was reached at the end of the diafiltration, and slightly higher than expected losses of 19% (vs. 13% predicted) were observed (Fig. 6A). The difference between the predicted and experimental purity can be explained by the stabilization of the system at the beginning of the diafiltration with small increase in purity observed between the diafiltration volume 0 and 1 (3% increase in purity instead of 7–8% expected), followed by a linear increase between 1 and 4.8 (Fig. 6A). By extrapolating the results, a purity of 85% could be obtained between 7 and 8 diafiltration volumes with an estimated 30% loss of (CoAS)_2_ content. Overall, the best system to purify (CoAS)_2_ from ATP, ADP and AMP is using a GK membrane at a TMP of 4 bars with a total compounds concentration solution of 5.19 g L^−1^. Using these conditions, we were able to isolate 365 mg of (CoAS)_2_ at a purity of 68% from a 225 mL crude solution. It has to be noted that these results can be greatly improved, especially in terms of productivity and purity, by using an experimental setup that would allow a larger membrane surface.

## Discussion

The use of cross-flow diafiltration for the purification of (CoAS)_2_ appeared to be a very good alternative solution to the existing ion exchange chromatography methodology. The membrane screening, evaluation and implementation of the GK membrane allowed us to show the high potential of this process, but it also provided us with data that can allow to confirm if the economy linked to this cofactor can be reversed or not. In the best conditions of our system, the productivity of our diafiltration system is 40.5 mg of 68% pure (CoA)_2_ per hour. Although this number seems to be quite small, it is important to note that the scale up possibilities are still to be explored and that this technology is already use in the industry (e.g. for water treatment). Indeed, by simply using a larger membrane surface, we can reach better productivity. The limits of this approach lie in the fact that it is necessary to have an adapted equipment that can handle large membrane surface and that the manufacturer produce large surface. In the case of GK membrane from Suez Water Technologies & Solutions, the larger commercial surface is 33.8 m^2^ in a spiral folding. It has both advantages to have a large active area coupling with a minimal carter size (101 cm high carter with a 20.1 cm diameter). Using such membrane would allow to reach a productivity of 174 g of 85% pure (CoAS)_2_ per cm consider that we have comparable flux that the one observed in our study which used plane GK membrane. Even if we consider a bad scenario with a flux divided by a factor 2 because of the folding, the productivity would still be very high with 87 g per hour. This will therefore significantly drop the production cost of (CoAS)_2_, will allow its production at an industrial scale and so will unlock the development of CoA-dependent biocatalytic systems.

**Figure 7:**
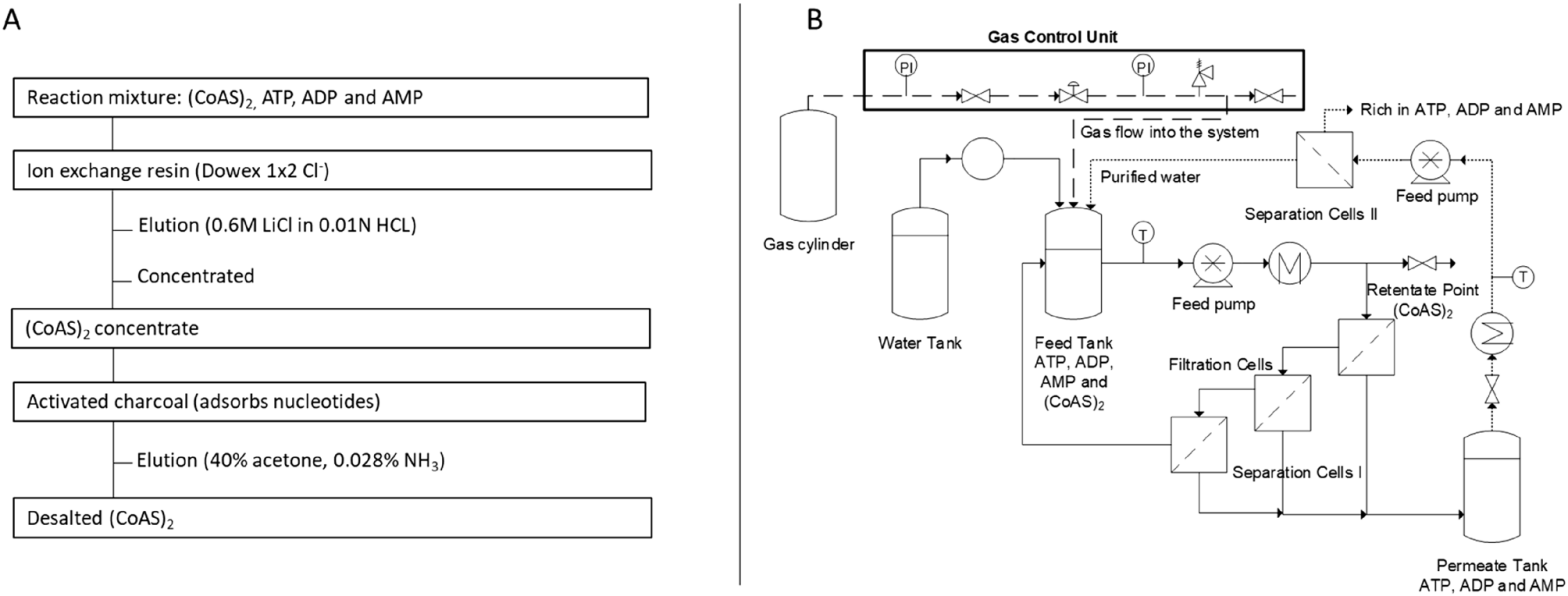
(**A**) Purification of (CoAS)_2_ through chromatography-charcoal adsorption/desorption strategy. (**B**) Purification of (CoAS)_2_ through cross-flow dia-filtration strategy.

Furthermore, the environmental impact of both chromatography and membrane methods was assessed through the measurement of the process mass intensity (PMI). In the chromatography and charcoal adsorption/desorption system, after binding (CoAS)_2_, ATP, ADP and AMP onto DOWEX anion exchange resin, increasing concentrations of LiCl are used to elute ATP, ADP, AMP and (CoAS)_2_, the latter being eluted by 600 mM LiCl. The volume of the solution is then reduced by evaporation of the water and (CoAS)_2_ adsorbed on activated charcoal. Excess LiCl is removed by rinsing the activated charcoal with deionized water, then (CoAS)_2_ is eluted with a 40% acetone solution containing 0.028% ammonia. The solution is then concentrated under reduced pressure and the residual water removed by freeze-drying to yield the desired product. It has to be noted that this chromatographic method (without considering the charcoal step) is less time consuming—5 h of elution—and that smaller losses were observed (~ 8%). For this method, a 650 × 25 mm DOWEX 1 × 2 anion exchange column, 53.5 g of LiCl, 20 g of activated charcoal, 4 L of water and 200 mL of acetone containing 0.028% ammonia are necessary to isolate 1 g of (CoAS)_2_ at a 82% purity (Fig. 6). Whereas in the membrane-based process described in this study, the same quantity of (CoAS)_2_ can be obtain with the same level of purity while avoiding the use of any other—toxic/hazardous—reagent. It is also noteworthy to mention that the diafiltration process can be readily optimized to recycle the water, as shown in Fig. 7 (dashed lines).

In order to quantitively assess the difference between the two methods in terms of sustainability, the PMI and the simple PMI (sPMI which excludes solvents) for both systems were calculated using the following formulas:

1 $$PMI = \frac{{\sum m\left( {total\, compounds} \right) + \sum m\left( {solvents} \right)}}{{m\left( {desired\, product} \right)}}$$

2 $$sPMI = \frac{{\sum m\left( {total\, compounds} \right) }}{{m\left( {desired\, product} \right)}}$$

In order to have more readable data, the quantity of water was not included in the calculations since it is in large excess compare to the rest of the compounds and is not differentiating (4 Liters of water is necessary in both system in order to obtain 1 g of the desired product). Thereby, the PMIs were calculated to be 372 for the classic chromatography and 3.55 for the diafiltration, meaning that 100 times more chemical waste by weight is produced using the chromatographic method compared to the diafiltration in order to purify the same amount of (CoAS)_2_. Even by removing the solvent from the calculation (sPMI), we observe a ratio of 14 which is considerable as we must do necessary efforts towards environmentally friendly processes (Table 3).

**Table 3:** The calculations of the PMI and sPMI of classic chromatography and diafiltration.

	Input compounds		Masse (g)	Output compound	Masse (g)	PMI	sPMI
Classic chromatography	Reagents	ATP	0.287	(CoAS)_2_	1	372.00	58.40
		ADP	1.22				
		AMP	0.087				
		(CoAS)_2_	1.095				
		HCl	1.93				
		Hydroxylamine	0.784				
		LiCl	53				
	Solvents	Acetone	313.6				
		Water	4000				
Diafiltration	Reagents	ATP	0.38	(CoAS)_2_	1	3.55	3.55
		ADP	1.61				
		AMP	0.115				
		(CoAS)_2_	1.45				
	Solvent	Water	4000				

Finally, in addition to be sustainable, integrated (purification and desalting at the same time) and to have great scale up potential, this strategy allows to unlock the access to (CoAS)_2_ at a multigram scale and therefore open the road to the development of CoA dependent biocatalytic systems until then little developed because of its prohibitive price. Such development of alternative purification methods is crucial in order to reveal the full potential of biocatalysis at larger scale.

## Methods

### Chemicals

Model solution and calibration curves were obtained with AMP (TCI, > 98%), ADP (TCI, > 98%), ATP (Acros Organics, 98%) and (CoAS)_2_ (Sigma-Aldrich, 85%). The model solution for the membranes screening was composed of AMP (0.04 g L^−1^), ADP (0.52 g L^−1^), ATP (0.15 g L^−1^) and (CoAS)_2_ (0.29 g L^−1^) with a global concentration of 1.00 g L^−1^. The stock solution from the (CoAS)_2_ enzymatic synthesis was composed of 80 mM of KCl, 40 mM of MgCl_2_, 200 mM of Tris and 19.0 g L^−1^ of (CoAS)_2_, 5.0 g L^−1^ of ATP, 21.0 g L^−1^ of ADP and 1.5 g L^−1^ of AMP^32^. The latter was diluted in order to (i) obtain different concentration solutions and (ii) assume a negligible concentration polarization.

### HPLC/MS method

The HPLC analyzes were performed on a Dionex Ultimate 3000 (Dionex Corporation, USA) equipped with a diode array detector (260 nm) and an Acclaim Polar Advantage II C18 column 150 mm × 4.6 mm × 3 µm (Thermo scientific). The temperature and the injection volume were 25 °C and 15 µL, respectively. Monosodium phosphate (NaH_2_PO_4_, 50 mM, solvent A) and acetonitrile (solvent B) were used as mobile phase with a constant flow rate of 1 mL.min^−1^. Gradient was: 1.2% (B) held 2.5 min, 1.2–7.2% (B) in 5 min, 7.2–11.2% (B) in 2.5 min, 11.2–20% (B) in 3 min, 20–30% (B) in 0.5 min, 30% (B) held 2.5 min, 30% back to 1.2% (B) in 0.5 min, 1.2% (B) held 3.5 min. Samples were filtered on 0.2 µm regenerated cellulose prior injection. Retentions times were as follows: ATP 2.6 min, ADP 3.1 min, AMP 6.1 min, (CoAS)_2_ 11.6 min.

### Membranes

Seven commercial flat sheet membranes were selected and tested to purify (CoAS)_2_: GE and GK from Suez Water Technologies & Solutions (USA), NP010 from Microdyn-Nadir (Germany), GR95PP from Alfa Laval (Sweden) and HYDRACoRe50-PS 7450 and HYDRACoRe70pHT Series 7470 PHT from Hydranautics-Nitto (USA). Most of them were designed for ultrafiltration processes, and the other for nanofiltration, depending on their molecular weight cut-off (MWCO). Tables 4 summarize the properties of the applied membranes and their field of application (given by manufacturers).

**Table 4:** Overview of the selected membranes and their specifications.

Membrane	MWCO (Da)	Water permeabilities (L h^−1^ m^−2^)	Type	T_max_ (°C)	pH	P_max_ (bar)	Material
GE	1000 for PEG*	3.06	UF	70	1–11	40	Modified polyamide
GK	3500 for PEG*	8.16	UF	70	1–11	27	Modified polyamide
NP010	1000	12.96	NF	95	0–14	40	PES**
GR95PP	2000	11.05	UF	75	1–13	10	PES**
HYDRACoRe50-PS 7450	1000	3.82	UF	60	2–11	41	Sulfonated PES**
HYDRACoRe70pHT Series 7470 PHT	720	2.79	NF	70	1–13,5	41	Sulfonated PES**

*PEG: Poly(ethylene glycol), **PES: Polyethersulfone.

### Experimental filtration set up

Filtration experiments were carried out in a METCell filtration device, from Evonik Industries (UK), in cross-flow operating mode. METCell is a stainless-steel system, designed to test flat sheet membranes from microfiltration to reverse osmosis, which is regulated with a gas control unit (inert N_2_ gas, from 1 to 65 bar). In cross-flow configuration, three filtration cells supported by porous discs (effective area of 39 cm^2^, 13 cm^2^ each), a feed tank (total volume of 600 mL) and a recirculation pump (AEG, 1.2 L/min), compose the system. Three flat sheet membranes can be used at the same time to allow (i) a screening (in total recirculation) to test different membranes (NB: the permeate flow being negligible compared to the retentate flow, the membranes can be considered as being parallel), (ii) a diafiltration or a concentration to increase membrane area. A high-performance liquid chromatography (HPLC) pump (Model 306, Gilson) was added to the system to enable total recirculation and diafiltration mode. A cooling system (Minichiller) and a balance (0.1 g, Sartorius) were also used to regulate the temperature and measure the permeate rate, respectively.

### Filtration parameters

Membrane filtration was defined by some parameters based on Darcy’s law (Eq. 3), with J_v_ (L h^−1^ m^−2^) the permeate flux, L_p_ (L h^−1^ m^−2^ bar^−1^) the solvent permeability and TMP (bar) the transmembrane pressure.

3 $$J_{v} = L_{p} \times TMP$$

For solvent (here pure water), the permeate flux was proportional to TMP, based on Darcy’s law, with J_0_ (m s^−1^) the solvent permeate flux, TMP (Pa) the transmembrane pressure, µ (Pa.s) the solvent dynamic viscosity and R_m_ (m^−1^) the intrinsic membrane resistance.

4 $$J_{0} = \frac{TMP}{{\mu \times R_{m} }}$$

As the solvent dynamic viscosity (µ) depends on temperature, during filtration, after evaluating the solvent temperature, the permeate flux was corrected with solvent dynamic viscosity at 20 °C. Then, solvent permeability (L_p_) was calculated to evaluate membrane performance and fouling.

During filtration, J_v_ was calculated by monitoring the permeate volume V_p_ (L) and applying Eq. (5), where A (m^2^) is the membrane filtration effective area and Δt (h) the filtration time.

5 $$J_{v} = \frac{{V_{p} }}{A \times \Delta t}$$

Membrane performance was evaluated by J_v_ and the rejection R_i_ (%). Rejection of the compound i was calculated using C_p,i_ and C_r,i_, the concentrations (g L^−1^) of i in the permeate and the retentate, respectively.

6 $$R_{i} = \left( {1 - \frac{{C_{p,i} }}{{C_{r,i} }}} \right) \times 100$$

During diafiltration, losses and purities were calculated using Eqs. (7) and (8), respectively, at each diafiltration volume (DV, one diafiltration volume corresponding to the initial feed volume). C_f,i,0_ is the concentration in the feed of solute i, at t = 0 min. C_r,i,t_ , C_r,j,t_ and C_r,k,t_ are the concentrations in the retentate of solutes i, j, k during the diafiltration experiments.

7 $$Loss_{i} = \frac{{\left( {C_{f,i,0} - C_{r,i,t} } \right)}}{{C_{f,i,0} }} \times 100$$

8 $$Purity_{i} = \frac{{C_{r,i,t} }}{{\left( {C_{r,i,t} + C_{r,j,t} + C_{r,k,t} } \right)}} \times 100$$

During diafiltration, losses and purities can be predict using the following general formula.

9 $$\frac{Ct}{{Co}} = \exp \left\{ { - \frac{{\left( {1 - Ri} \right) Vt}}{Vo} } \right\}$$

were C_0_, C_t_, R, V_0_ and V_t_ are the concentration of the studied chemical at the initial time, the concentration of the studied chemical at any time, the retention rate for the compound i, the volume of the solution at the initial time and the volume of the solution at any time, respectively.

### Membrane screening

Selected membranes were placed in the METcell device and washed according to the manufacturer procedure. Afterward, membranes were compacted with reverse osmosis water, in total recirculation mode, at different pressure levels for 15 min until the chosen maximal pressure for each membrane was attained. The compaction step was validated once permeate flux reached stability at maximal pressure. Flux was then monitored using ultra-pure water, in total recirculation mode, for each membrane, at different transmembrane pressures and at room temperature (25 °C). According to Eq. (3), membrane permeabilities were then determined for each membrane. The feed tank was then filled with 700 mL of the model solution. Permeate flux was evaluated at the studied pressure levels using Eq. (5), in total recirculation mode. Once permeate flux reached steady state, for each transmembrane pressure, retentate and permeate samples were collected for HPLC analyzes. Membrane rejection was calculated for each solute, each transmembrane pressure and each membrane, according to Eq. (6). These experiments allowed to determine the best membrane and pressure to use to purify the (CoAS)_2_.

### Optimization of starting solution concentration, with selected membrane

To limit water consumption used for dilution, four different concentrations of solution (obtained from the stock solution) were tested: 1.51 g L^−1^, 2.43 g L^−1^, 5.19 g L^−1^ and 10.71 g L^−1^ of total compounds (all the solutions are with the ATP/ADP/AMP/(CoAS)_2_ ratio at 3.3/14/1/12.6). Membrane performances of selected membranes were quantified (in total recirculation mode) in duplicate, after membrane washing/compaction and membrane water permeability evaluation, by determining permeate flux (Eq. 5) and solutes rejections (Eq. 6).

### Diafiltration experiments

In order to eliminate ATP, ADP and AMP via membrane filtration, a diafiltration was operated. The selected membrane was placed in the METcell system (3 × 13 cm^2^), then washed and compacted with reverse osmosis water, in total recirculation mode, until attaining stable permeate flux. Water permeability, with ultrapure water, was then evaluated by measuring permeate flux at different pressures. After filling the feed tank with 200–250 mL of solution at the optimized concentration (obtained from stock solution), the system was set up at selected pressure at 25 °C, in total recirculation. The diafiltration, in continuous mode, started when ultrapure water was added to the feed via HPLC pump, and the permeate collected in vessel. At each diafiltration volume (DV), samples of retentate were collected for HPLC analyzes. Diafiltration was carried out until desired DV (based on Eq. 9) and in duplicate. Afterwards, pressure was released to reach atmospheric pressure and the retentate solution was recovered. Membrane fouling was evaluated by measuring water permeability after the process.

## Supplementary Information

Supplementary Information.

## Data Availability

The datasets used and/or analyzed during the current study available from the corresponding author on reasonable request.
