# Supplementary material for: Unlocking the access to oxidized coenzyme A via a single-step green membrane-based purification
Source: Sci Rep. 2022 Jul 29;12:12991. doi: 10.1038/s41598-022-17250-8 (PMC9338019; doi:10.1038/s41598-022-17250-8)

**SUPPORTING INFORMATION**

Unlocking the access to oxidized coenzyme A *via* a single-step green membrane-based purification

Louis M. M. Mouterde ^*a^, Gaelle Willig ^a^, Maxime M. J. Langlait ^a^, Fanny Brunois ^a^, Morad Chadni ^a^, Florent Allais ^a^,

URD Agro-Biotechnologies Industrielles (ABI), CEBB, AgroParisTech, 51110, Pomacle, France

Phone +33 3 52 62 04 65, E-mail [louis.mouterde@agroparistech.fr](mailto:louis.mouterde@agroparistech.fr)

*Author to whom correspondence should be addressed

**Contents**

[Retention profile of the selected membranes towards model solution S-3](#_Toc102573210)

[Effect of pressure (TMP) on permeate flux (J_v_) for membranes screened with model solution. () NP010, () GE, () 7450, (x) 7470 PHT S-4](#_Toc102573211)

[Effect of pressure (TMP) on permeate flux (J_v_) for membranes screened with model solution. (+) GK, () GR95PP S-4](#_Toc102573212)

[Effect of pressure (TMP) on the compounds retention for NP010 membranes with model solution. (x) (CoAS)_2_, () ATP, () ADP, () AMP S-5](#_Toc102573213)

[Effect of pressure (TMP) on the compounds retention for GE membranes with model solution. (x) (CoAS)_2_, () ATP, () ADP, () AMP S-5](#_Toc102573214)

[Effect of pressure (TMP) on the compounds retention for 7450 membranes with model solution. (x) (CoAS)_2_, () ATP, () ADP, () AMP S-6](#_Toc102573215)

[Effect of pressure (TMP) on the compounds retention for 7470 PHT membranes with model solution. (x) (CoAS)_2_, () ATP, () ADP, () AMP S-6](#_Toc102573216)

[Effect of pressure (TMP) on the compounds retention for GK membranes with model solution. (x) (CoAS)_2_, () ATP, () ADP, () AMP S-7](#_Toc102573217)

[Effect of pressure (TMP) on the compounds retention for GR95PP membranes with model solution. (x) (CoAS)_2_, () ATP, () ADP, () AMP S-7](#_Toc102573218)

[Effects of 1.51 g L^-1^ concentration on the compounds retention as a function of transmembrane pressure (TMP). (x) (CoAS)_2_, () ATP, () ADP, () AMP (experiments were carried on in duplicate) S-8](#_Toc102573219)

[Effects of 2.43 g L^-1^ concentration on the compounds retention as a function of transmembrane pressure (TMP). (x) (CoAS)_2_, () ATP, () ADP, () AMP (experiments were carried on in duplicate) S-9](#_Toc102573220)

[Effects of 5.19 g L^-1^ concentration on the compounds retention as a function of transmembrane pressure (TMP). (x) (CoAS)_2_, () ATP, () ADP, () AMP (experiments were carried on in duplicate) S-9](#_Toc102573221)

[Effects of 10.71 g L^-1^ concentration on the compounds retention as a function of transmembrane pressure (TMP). (x) (CoAS)_2_, () ATP, () ADP, () AMP (experiments carried on in duplicate) S-10](#_Toc102573222)

[Evolution of (CoAS)_2_ purity and loss as a function of diafiltration volume (DV) during dia-ultrafiltration process with 5.19 g L^-1^ solution (experiments were carried on in duplicate). Trend lines for purity and loss were applied to linear part S-11](#_Toc102573223)

[Evolution of (CoAS)_2_ purity and loss as a function of diafiltration volume (DV) during dia-ultrafiltration process with 5.19 g L^-1^ solution (experiments were carried on in duplicate) S-11](#_Toc102573224)

[Evolution of permeate flux (Jv) and diafiltration volume (DV) as a function of time, with 5.19 g L^-1^ solution (experiments were carried on in duplicate) S-12](#_Toc102573225)

[Dia-ultrafiltration alimentation chromatogram for 5.19 g L^-1^ S-12](#_Toc102573226)

[Dia-ultrafiltration retentate at DV2 chromatogram, for 5.19 g L^-1^ S-13](#_Toc102573227)

[Dia-ultrafiltration final retentate chromatogram, for 5.19 g L^-1^ S-13](#_Toc102573228)

[Dia-ultrafiltration final permeate chromatogram, for 5.19 g L^-1^ S-14](#_Toc102573229)

# Retention profile of the selected membranes towards model solution

| **Membrane** | **Pression (bar)** | **Retention of (CoAS)_2_ (%)** | **Retention of ATP (%)** | **Retention of ADP (%)** | **Retention of AMP (%)** |
| --- | --- | --- | --- | --- | --- |
| NP010 | 10 | 95.26 | 91.87 | 88.53 | 86.78 |
|  | 20 | 100 | 91.78 | 87.57 | 85.17 |
|  | 30 | 100 | 93.72 | 89.73 | 86.69 |
|  | 35 | 100 | 94.27 | 90.27 | 86.94 |
| GE | 10 | 97.52 | 100 | 96.68 | 91.68 |
|  | 20 | 98.34 | 100 | 97.23 | 96.78 |
|  | 30 | 94 | 91.98 | 90.59 | 90.57 |
|  | 35 | 95.20 | 93.23 | 91.43 | 91.12 |
| GK | 4 | 97.44 | 62.30 | 46.64 | 7.55 |
|  | 6 | 97.22 | 66.20 | 52.07 | 12.78 |
|  | 8 | 97.35 | 67.46 | 54.05 | 12.80 |
|  | 10 | 96.72 | 67.84 | 55.48 | 15.33 |
| HydraCore50-PS 7450 | 10 | 100 | 99.83 | 99.93 | 99.28 |
|  | 20 | 100 | 100 | 100 | 99.60 |
|  | 30 | 100 | 100 | 100 | 99.58 |
|  | 35 | 100 | 100 | 100 | 99.70 |
| HydraCore70pHT Series 7470PHT | 10 | 99.26 | 98.93 | 99.73 | 99.45 |
|  | 20 | 100 | 100 | 100 | 99.72 |
|  | 30 | 100 | 100 | 100 | 99.81 |
|  | 35 | 100 | 100 | 100 | 99.84 |
| GR95PP | 4 | 65.38 | 31.18 | 22.85 | 12.87 |
|  | 6 | 73.11 | 41.94 | 32.33 | 18.44 |
|  | 8 | 75.01 | 48.90 | 39.49 | 24.63 |
|  | 10 | 75.44 | 51.52 | 42.33 | 27.26 |

# Effect of pressure (TMP) on permeate flux (J_v_) for membranes screened with model solution. () NP010, () GE, () 7450, (x) 7470 PHT

# Effect of pressure (TMP) on permeate flux (J_v_) for membranes screened with model solution. (+) GK, () GR95PP

# Effect of pressure (TMP) on the compounds retention for NP010 membranes with model solution. (x) (CoAS)_2_, () ATP, () ADP, () AMP

# Effect of pressure (TMP) on the compounds retention for GE membranes with model solution. (x) (CoAS)_2_, () ATP, () ADP, () AMP

# Effect of pressure (TMP) on the compounds retention for 7450 membranes with model solution. (x) (CoAS)_2_, () ATP, () ADP, () AMP

# Effect of pressure (TMP) on the compounds retention for 7470 PHT membranes with model solution. (x) (CoAS)_2_, () ATP, () ADP, () AMP

# Effect of pressure (TMP) on the compounds retention for GK membranes with model solution. (x) (CoAS)_2_, () ATP, () ADP, () AMP

# Effect of pressure (TMP) on the compounds retention for GR95PP membranes with model solution. (x) (CoAS)_2_, () ATP, () ADP, () AMP

Effects of concentrations on permeate flux (Jv) as a function of transmembrane pressure (TMP). () 10.71 g L^-1^, () 1.51 g L^-1^, (x) 2.43 g L^-1^, () 5.19 g L^-1^ (experiments were carried on in duplicate)

# Effects of 1.51 g L^-1^ concentration on the compounds retention as a function of transmembrane pressure (TMP). (x) (CoAS)_2_, () ATP, () ADP, () AMP (experiments were carried on in duplicate)

# Effects of 2.43 g L^-1^ concentration on the compounds retention as a function of transmembrane pressure (TMP). (x) (CoAS)_2_, () ATP, () ADP, () AMP (experiments were carried on in duplicate)

# Effects of 5.19 g L^-1^ concentration on the compounds retention as a function of transmembrane pressure (TMP). (x) (CoAS)_2_, () ATP, () ADP, () AMP (experiments were carried on in duplicate)

# Effects of 10.71 g L^-1^ concentration on the compounds retention as a function of transmembrane pressure (TMP). (x) (CoAS)_2_, () ATP, () ADP, () AMP (experiments were carried on in duplicate)

# Evolution of (CoAS)_2_ purity and loss as a function of diafiltration volume (DV) during dia-ultrafiltration process with 5.19 g L^-1^ solution (experiments were carried on in duplicate). Trend lines for purity and loss were applied to linear part

# Evolution of (CoAS)_2_ purity and loss as a function of diafiltration volume (DV) during dia-ultrafiltration process with 5.19 g L^-1^ solution (experiments were carried on in duplicate)

# Evolution of permeate flux (Jv) and diafiltration volume (DV) as a function of time, with 5.19 g L^-1^ solution (experiments were carried on in duplicate)

# Dia-ultrafiltration alimentation chromatogram for 5.19 g L^-1^


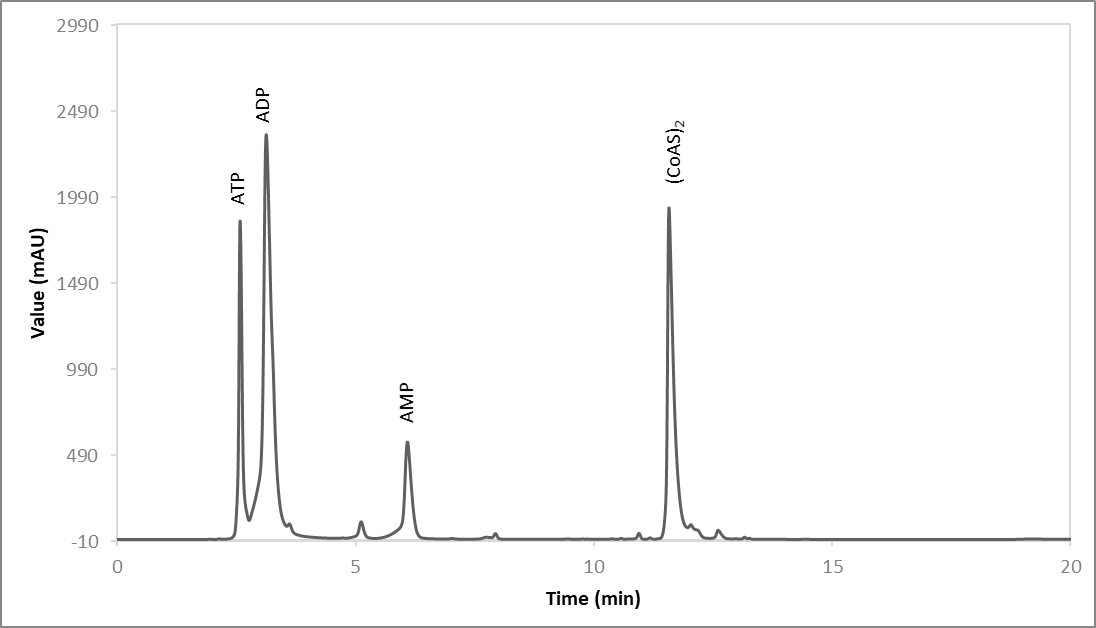


# Dia-ultrafiltration retentate at DV2 chromatogram, for 5.19 g L^-1^


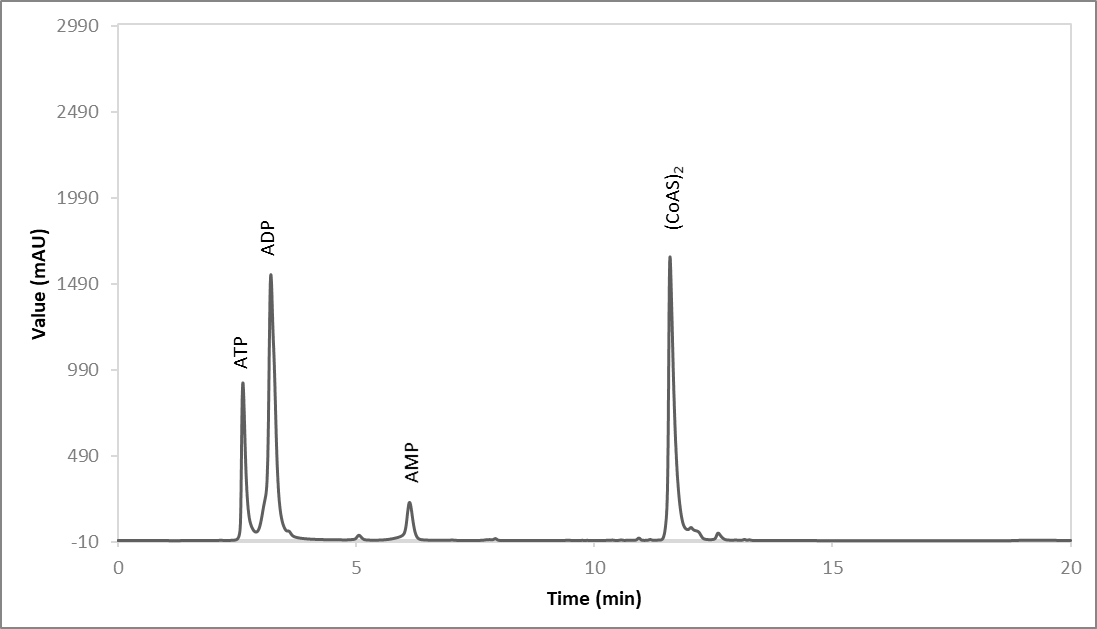


# Dia-ultrafiltration final retentate chromatogram, for 5.19 g L^-1^


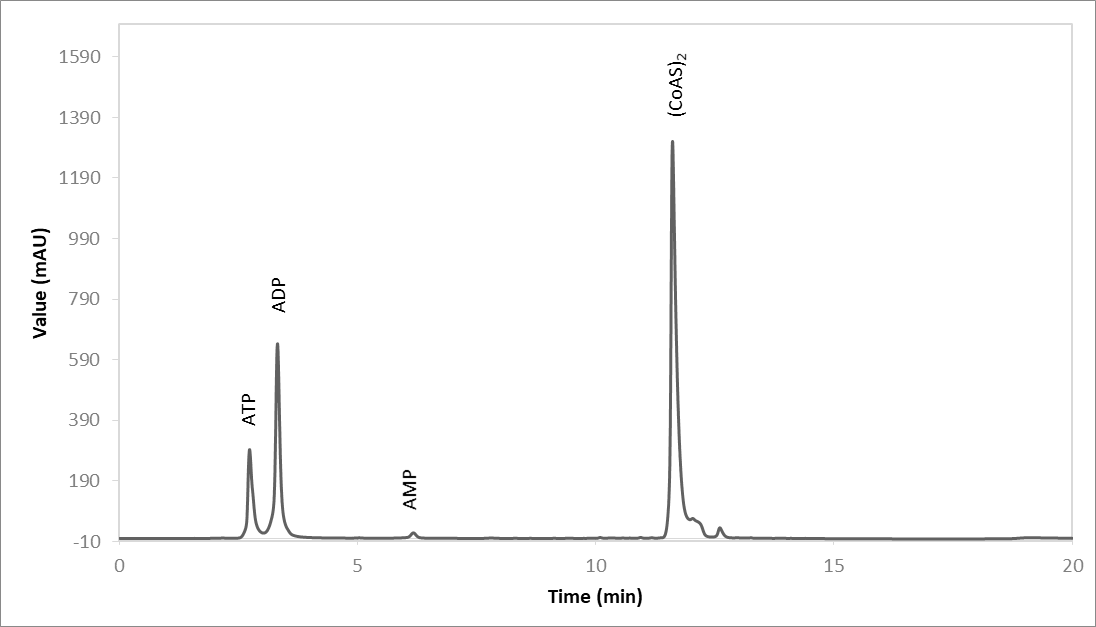


# Dia-ultrafiltration final permeate chromatogram, for 5.19 g L^-1^


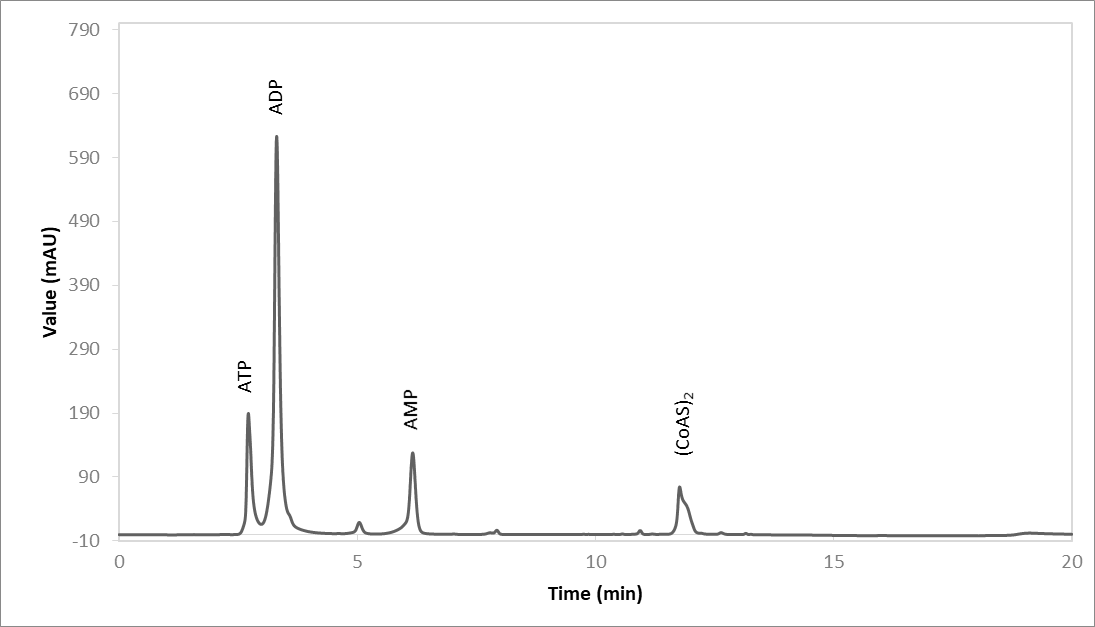

Supplement: Supplementary file 1 — Supplementary Information. [file 41598_2022_17250_MOESM1_ESM.docx]
